# Supplementary material for: Marker-Assisted Selection of Jacalin-Related Lectin Genes OsJRL45 and OsJRL40 Derived from Sea Rice 86 Enhances Salt Tolerance in Rice
Source: Int J Mol Sci. 2024 Oct 10;25(20):10912. doi: 10.3390/ijms252010912 (PMC11507410; doi:10.3390/ijms252010912)
Supplement: Supplementary file 1 [file ijms-25-10912-s001.zip › ijms-3234811-supplementary.pdf]

Marker-Assisted Selection of Jacalin-Related Lectin Genes *OsJRL45* and *OsJRL40* Derived from Sea Rice 86 Enhances Salt Tolerance in Rice

Xiaolin Yin, Qinmei Gao, Feng Wang, Weihao Liu, Yiting Luo, Shuixiu Zhong, Jiahui Feng, Rui Bai, Liangbi Chen, Xiaojun Dai *, Manzhong Liang *

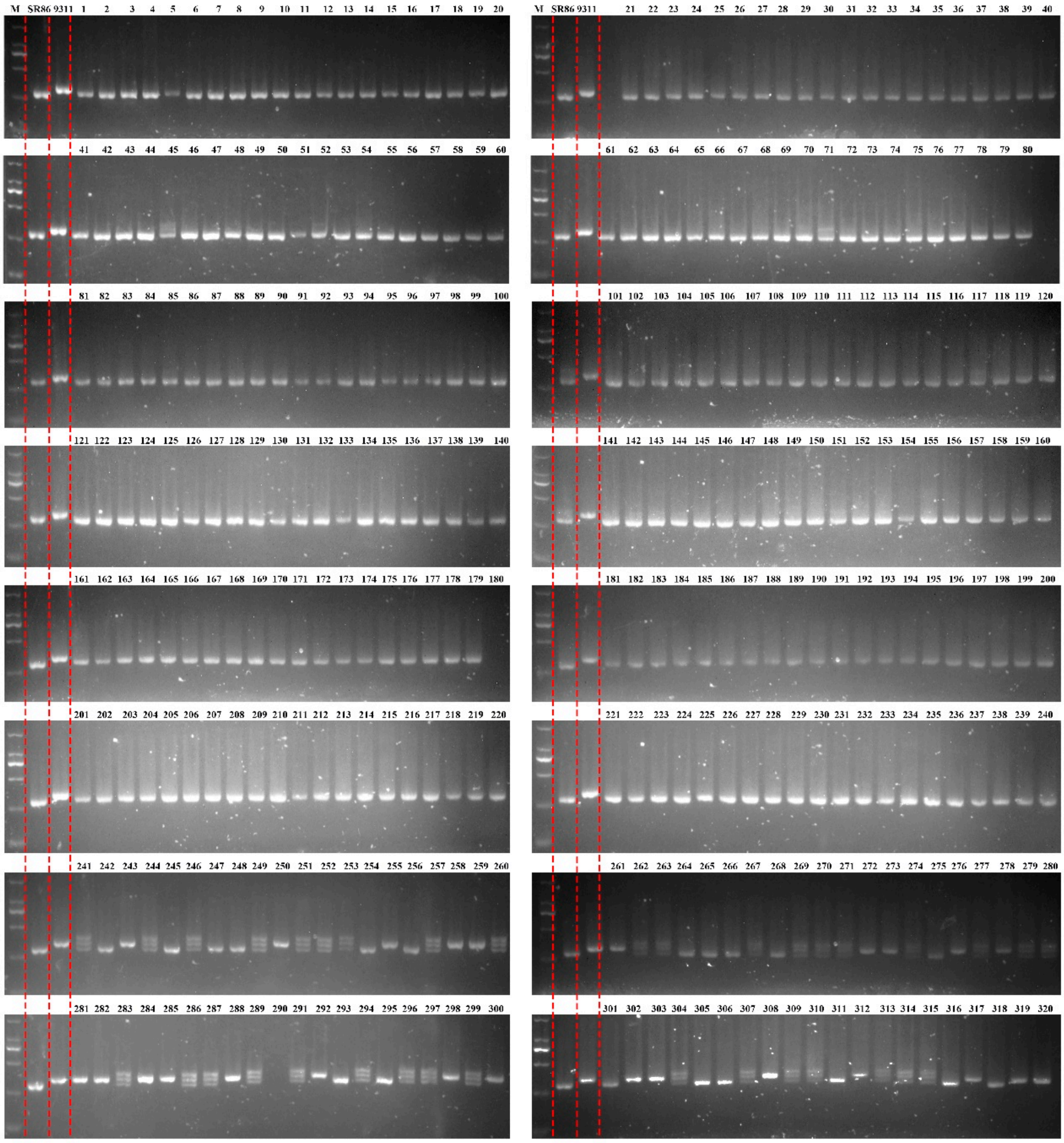

Supplementary Figure S1. 2% agarose gel electrophoresis of 320 SR86×9311 RILs. Lanes M, SR86 and 9311 (indicated by the red dotted lines) represent DNA size marker, SR86, and 9311, respectively. From left to right (lanes 1–320) 320 SR86×9311 RILs.

Supplementary Table S1 Primers Constructed by Vector.

Name	Primer sequence (5'-3')
1300-OsJRL45-F	GGATCTTCCAGAGATGGATCCACTTCTCCACACTAGAAGGGATACCC
1300-OsJRL45-R	CTGCCGTTCGACGATAAGCTTCTACTCACTCTCGTGTTTGTGTGTGT
1300-OsJRL40-F	GGATCTTCCAGAGATGGATCCTGCAAATATAAAAATATTTATGTTATGCTCA
1300-OsJRL40-R	CTGCCGTTCGACGATAAGCTTTTAGCTCTGGGGACTGACATAGACC
AD-OsJRL45CDS-F	GGATCTTCCAGAGATGAATTCATGGGAAACTTCTCCGGTGC
AD-OsJRL45CDS-R	CTGCCGTTCGACGATGGATCCGATTGGGCTAATGTAGACTCCGA
BD-OsJRL40CDS-F	GGATCTTCCAGAGATGAATTCATGGAGAGCAACAACAACAGCA
BD-OsJRL40CDS-R	CTGCCGTTCGACGATCTGCAGGCTCTGGGGACTGACATAGACTCC
OsJRL45CDS-YFP^{C}-F	CATCGAGGACGCCGGCGGATCCATGGAGAGCAACAACAACAGCAG
OsJRL45CDS-YFP^{C}-R	AAGCTCTGCAGGTCGACTCTAGATTAGCTCTGGGGACTGACATAGAC
OsJRL40CDS-YFP^{N}-F	CATCGAGGACGCCGGCGGATCCATGGAGAGCAACAACAACAGCAG
OsJRL40CDS-YFP^{N}-R	AAGCTCTGCAGGTCGACTCTAGATTAGCTCTGGGGACTGACATAGAC

Supplementary Table S2 Quantitative Primers.

Name	Primer sequence (5'-3')
Osactin-F	CAATGTGCCAGCTATGTATGTCGCC
Osactin-R	TTCCCGTTCAGCAGTGGTAGTGAAG
OsJRL45-F	TGAACAGGTGACTTCGGTGG
OsJRL45-R	ATGGTCCGTATGTCTTGCCG
OsJRL40-F	CAGCAACATGGAGGACGTGA
OsJRL40-R	CATGGATCGTTGCTTACCTCA

Supplementary Table S3 Molecular Markers Primers.

Name	Primer sequence (5'-3')
BJ-F	GCAAGACATACGGACTGTTGGAAAT
BJ-R	ATCGGGTTCAGTTGGAGAGCG
